# Programming of Mice Circadian Photic Responses by Postnatal Light Environment

**DOI:** 10.1371/journal.pone.0097160

**Published:** 2014-05-19

**Authors:** Elisabeth Brooks, Dhruval Patel, Maria Mercè Canal

**Affiliations:** Faculty of Life Sciences, University of Manchester, Manchester, United Kingdom; University of Alabama at Birmingham, United States of America

## Abstract

Early life programming has important consequences for future health and wellbeing. A key new aspect is the impact of perinatal light on the circadian system. Postnatal light environment will program circadian behavior, together with cell morphology and clock gene function within the suprachiasmatic nucleus (SCN) of the hypothalamus, the principal circadian clock in mammals. Nevertheless, it is still not clear whether the observed changes reflect a processing of an altered photic input from the retina, rather than an imprinting of the intrinsic molecular clock mechanisms. Here, we addressed the issue by systematically probing the mouse circadian system at various levels. Firstly, we used electroretinography, pupillometry and histology protocols to show that gross retinal function and morphology in the adult are largely independent of postnatal light experiences that modulate circadian photosensitivity. Secondly, we used circadian activity protocols to show that only the animal's behavioral responses to chronic light exposure, but not to constant darkness or the acute responses to a light stimulus depend on postnatal light experience. Thirdly, we used real-time PER2::LUC rhythm recording to show long-term changes in clock gene expression in the SCN, but also heart, lung and spleen. The data showed that perinatal light mainly targets the long-term adaptive responses of the circadian clock to environmental light, rather than the retina or intrinsic clock mechanisms. Finally, we found long-term effects on circadian peripheral clocks, suggesting far-reaching consequences for the animal's overall physiology.

## Introduction

The suprachiasmatic nucleus (SCN) in the hypothalamus organizes physiology and behavior of an individual in a 24 h fashion, and synchronizes them to the particular environment the animal lives in. For animals, synchronization to the environment is critical for survival, while for humans, it is key to avoid a number of physiological and mental health problems in the long run, as those experienced by shift-workers [1]. A crucial strategy to ensure full adaptation of an individual to its surroundings is to fine-tune this synchronization after birth, when the circadian system is not yet mature and can thus be shaped by experience. Several studies in the last few years have highlighted the fact that postnatal environment, and in particular light, exerts a major long-lasting influence on the individual's circadian system later in life [2]. This may also occur in preterm babies exposed to abnormal light environments in Neonatal Intensive Care Units, as this experience can affect their short-term recovery and growth [3], together with their long-term sleep patterns and neurodevelopment [4], [5]. Therefore, it is critical to determine the mechanisms behind early programming of the circadian system by light, as a first step towards uncovering the consequences of abnormal light exposure during development.

Rodents are born with an immature circadian system that will develop during the critical period of the first 3 postnatal weeks [6], making them an ideal model system to study the programming effects of environmental light on the developing circadian system. It is now known that the light environment (24-h light-dark cycles, constant light, constant darkness, photoperiod) that a rodent pup experiences during the critical period of development will impact its circadian behavior later in life [7], [8], [9], and that these behavioral changes correspond with alterations at the SCN level, including protein expression [7], [10], an altered number of neurons and glial cells [11], [12], and Per1 clock gene changes [13]. Furthermore, these behavioral and neurological alterations are independent of maternal circadian behavior [14], and are observed well into adulthood, long after the end of the critical period [15], suggesting these changes are long-lasting.

Nevertheless, it is not yet clear whether the observed changes within the SCN might in fact be reflecting a processing of an altered photic input from the retina. Here, we addressed the issue by systematically probing the circadian system at various levels: retina, SCN, peripheral tissues and circadian behavior. Our data reveal that postnatal light experience mainly targets the long-term adaptive responses of the circadian clock to environmental light, rather than the retina or the SCN clock's intrinsic mechanisms. Finally, and for the first time, we found long-lasting effects in circadian peripheral clocks, suggesting far-reaching consequences for the animal's overall physiology.

## Materials and Methods

### Animals

Pregnant C57BL/6J, CD1 and homozygous PER2::LUC knock-in mice [16] generated on a C57BL/6J background, bred and raised in our colony, were kept under a 24 h LD cycle with 12 h of light and 12 h of darkness (LD 12:12) until embryonic day 18 (E18). They were then placed in constant darkness (DD), constant light (LL) or LD to give birth. Mean light intensity at cage floor level was 330 µW/cm^2^ provided by LED light. Pups were raised in these light conditions from the day of birth (postnatal day 0, P0) to P21 then all placed in LD and weaned between P21 and P25. For all studies we used both male and female mice.

Both C57BL/6J and CD1 mice were used in the experiments testing the effect of environmental light on retinal function. CD1 (albino) mice are more sensitive to retinal damage by light [17], [18] and therefore they were used here as positive controls. In all subsequent experiments, and in order to investigate the direct effects of early light on the circadian pacemaker without masking from an altered/impaired retinal function, only C57BL/6J mice were used. Finally, PER2::LUC mice were used in the experiment investigating the effects of early light environment on clock gene expression.

### Ethics statement

All experimental procedures were conducted in accordance with the United Kingdom Animals (Scientific Procedures) Act 1986, and were approved by the University of Manchester's Animal Ethics Committee.

### Electroretinography

Electroretinography (ERG) experiments were conducted as previously described [19], [20]. Mice were dark-adapted overnight before beginning the ERG experiment. All work during this experiment was performed under dim red light (<0.2 µW/cm^2^) in the rest phase, between Circadian Time (CT) 2 and CT10 (where CT12 =  time of activity onset).

Dark-adapted irradiance responses were elicited by white flash stimuli from a xenon arc source (Cairn Research Ltd, Faversham, UK) reflected in a custom-made Ganzfeld dome and attenuated with neutral density filters (Edmund Optics, York, UK) to obtain irradiances ranging from −4.9 to 3.1 log10 µW/cm^2^. An electrically controlled mechanical shutter (Cairn Research Ltd) was used to apply a series of single 15 ms flashes, each starting 200 ms after sweep onset. The interval between stimuli ranged from 1.5 ms for the lowest irradiances to 30 s for the highest irradiances and the number of stimuli for each irradiance decreased from 30 to 6 as the irradiance increased. An average trace was obtained for each irradiance level. The a-wave (when present) was quantified from the trough to the baseline in the unfiltered traces. To measure the amplitude of the b-wave, the ERG wave was filtered by removing any signal below 5 Hz, thus removing the influence of oscillatory potentials. The b-wave amplitude was quantified by summing the absolute a-wave amplitude (unfiltered) and the b-wave amplitude when filtered. The implicit time for the a-wave was measured from the stimulus onset to the trough of the a-wave. The implicit time for the b-wave was measured from the stimulus onset to the peak of the b-wave.

Cone-driven activity (photopic ERG) was isolated by measuring the responses to a series of bright white flashes (Grass Model PS33 Photic Stimulator, Astro-Med, Inc., West Warwick RI, fitted with a 400 nm high pass filter, 10 µs duration, peak corneal irradiance 1 log10 µW/cm^2^) applied at a frequency of 0.75 Hz against a uniform white background light (metal halide source) of sufficient intensity (400 µW/cm^2^) to saturate rods but not cones. The background light was left on and the ERG was recorded continuously at 0.75 Hz for 20 minutes. Average waveforms were obtained for every 25 frames and filtered (low pass 5 Hz) to exclude oscillatory potentials. The peak of the b-wave was identified from the averaged traces and its amplitude and implicit time (from the start of the flash) were calculated.

### Pupillometry

Pupillary light reflex (PLR) was conducted as previously described [21], [22]. Mice were entrained to a 24 h LD cycle then dark-adapted for 1 to 1.5 hours prior to the beginning of the experiment. Measurements were taken between ZT5 and ZT8 (where ZT12 =  time lights off). Pupillary responses were elicited by applying a light stimulus (200 µW/cm^2^) provided by Xenon arc lamp (Cairn Research Ltd.) filtered with a 480 nm monochromatic interference filter (half bandwidth ≤10 nm) and transmitted to the integrating sphere using a quartz fibre optic. Consensual pupil constriction could then be monitored in the left eye by illuminating it with infrared (>900 nm) and recording it with a CCD camera fitted with a 140 mm lens in a parallel plane to the cornea. The eye was recorded for 2 s with no light stimulus, then for a further 58 s as the light stimulus was applied. Pupil area was measured in images captured from the videotaped records using VirtualDub (web-based software package, VirtualDub.org) and Matlab R2008a (The Mathworks, Cambridge, UK).

### Retinal histology

Eyes were first drop-fixed in 4% paraformaldehyde (PFA; Sigma-Aldrich, Dorset, UK, 2 days), then cryoprotected in a 30% sucrose solution (sucrose in phosphate buffered saline solution, Sigma-Aldrich, 2 days), and finally stored at −20°C until processed. Transverse sections (10 µm) stained in cresyl violet (Sigma-Aldrich) were observed under a microscope (Leica DM2000, Leica Microsystems). Photographs of at least 6 retinal sections per mouse were analyzed using ImageJ (NIH, USA) by measuring the width of the outer nuclear layer (ONL) at 6 different points of the retinal section and taking the average width per section.

### Behavioral analysis

Locomotor activity was recorded continuously by means of infrared activity meters placed outside the cage, and it was analyzed using the software package El Temps (A. Díez-Noguera, Universitat de Barcelona, Spain) as previously described [7]. For each light condition, we calculated the following characteristics of the circadian rhythm: period and percentage of variance explained by the highest peak (using the X^2^-periodogram), amplitude and power content of the first harmonic (using Fourier analysis), daily active phase duration and activity levels (using the mean waveform). The magnitudes of the phase shifts produced by the light pulses were assessed by drawing a line through the daily onsets for the 10 days before and the 10 days after treatment. The difference between the two fitted lines on the day after the light pulse was the value given to the phase shift.

### Immunohistochemistry

Immunohistochemistry was conducted as previously described [7], [10]. For primary antibodies we used cFOS (1:10000, rabbit polyclonal, Santa Cruz Biotechnology Inc., CA, US) and pERK (1:5000, rabbit monoclonal, Cell Signaling Technology, Beverly, MA, US).

Sections were observed under a microscope (Leica DM2000, Leica Microsystems). For cFOS-stained sections, two investigators naive to the experimental groups counted the number of positively-stained cells in the left and right SCN for each section. For pERK-stained sections, digital images were captured electronically and a line was drawn around the SCN boundaries using Image J (version 1.38, NIH, Bethesda, US). Another area close to the SCN was used to determine the background of staining. The background value was subtracted from the SCN value to obtain the normalized optical density of pERK staining for each SCN.

### Tissue culture

PER2::LUC mice kept under 24 h LD cycles were culled between ZT4-5. Tissue culture was conducted as previously described [23], [24]. Tissues (brain, heart, lung, liver and spleen) were microdissected and cultured for a minimum of 5 days. Bioluminescence recordings were measured at 10 minute intervals using a Lumicycle (Actimetrics, Wilmette, IL, USA). PER2::LUC rhythms were analyzed using a method based on Abe et al. [25]. Briefly, the data was detrended using a 24-hour running average and smoothed using a 3-hour running average of the detrended data. The 3-hour running average was used to calculate the phase, amplitude, period and damping rate of the rhythm. The phase was calculated using the first peak of the data. The amplitude of the rhythm was determined using the first two peaks and troughs of the data. The period was calculated by taking the average of the periods of the first three cycles. The amplitude of each peak and trough was taken and plotted on a graph to calculate the damping rate. A linear trendline with its equation was fitted to the data and the point at which the trendline crossed the x-axis (y = 0) was taken as the damping rate in days.

### Statistical analysis

Statistical analysis was carried out by means of various ANOVA of general linear models using SYSTAT (version 10, SPSS, Inc., Chicago, IL, USA); interactions between independent variables were included in the model. When a significant result was encountered, a Bonferroni post-hoc analysis, which corrected for multiple pairwise comparisons, was applied. All results are expressed as mean ± standard deviation unless otherwise stated.

## Results

### Effects of postnatal light environment on adult mouse retinal function and structure

Light is key to synchronize the internal clock to the external environment [26]. It is detected by rod and cone photoreceptors in the retina, which convey image-forming visual information [27], along with the intrinsically photosensitive retinal ganglion cells (ipRGCs), which express melanopsin and play an important role in circadian entrainment [28]. Light can however cause retinal damage, in particular, in albino animals, which lack pigment [29], [30]. Retinal damage occurs in stages, starting in the rods, followed by cones. Once damage in the outer segments has occurred, damage to the inner segments begins [31].

To test whether the response of retinal photoreceptors to light was affected by postnatal light environment, both in albino and pigmented animals, we raised CD1 and C57BL/6J mice in either LD, DD or LL from the day of birth (postnatal day 0, P0) until weaning (P21), and then kept them under 24 h LD cycles until P35-P55, when retinal function was assessed. We used dark-adapted electroretinography (ERG) to assess functionality of rods and cones (using the a-wave) and ON-bipolar cells (using the b-wave) [19], [20]. We isolated cone-driven activity by using the photopic ERG [32]. We also determined the non-image forming (ipRGC-dependent) visual response by measuring PLR [33].

**Figure 1:**
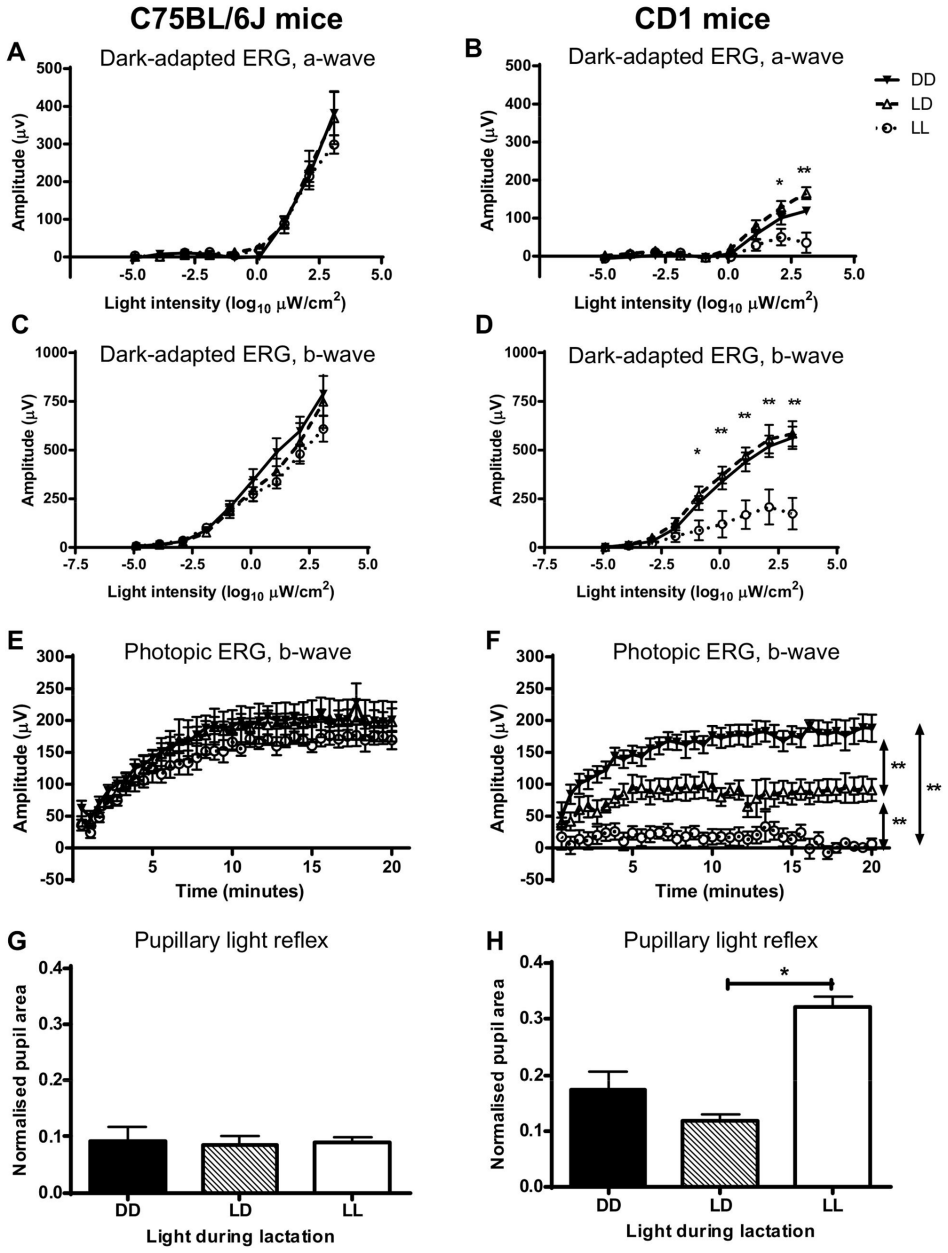
Postnatal light effects on retinal function in C57BL/6J and CD1 adult mice. Retinal function of adult mice reared in DD, LD or LL postnatally and later exposed to LD. (A, B) Representative dark-adapted ERG a-wave amplitude. (C, D) Amplitude of the dark-adapted ERG b-wave. (E, F) Amplitude of the photopic ERG b-wave. (G, H) Pupillary light reflex. ERG – electroretinogram; DD – constant darkness; LD –24 h light-dark cycles; LL – constant light. *P<0.05 DD, LD vs. LL; ** P<0.01 DD, LD vs. LL. Data are presented as mean ± s.e.m. (n = 7).

In C57BL/6J pigmented mice, we found no significant difference in either a-wave or b-wave amplitude of the dark-adapted ERG due to early light environment, while statistically significant differences due to intensity of the light stimulus were found, so that the higher the light intensity the higher the amplitude of the response (*F*_(8, 153)_ = 82.780, *p*<0.001 for a-wave and *F*_(8,153)_ = 65.523, *p*<0.001 for b-wave, Figs. 1A and 1C). We then measured the adaptation of cones to light using the photopic ERG and found no significant difference in the b-wave amplitude due to early light environment (Fig. 1E). Early light environment did not significantly affect the pupil responses of adult C57BL/6J mice to light (Fig. 1G). These data indicate that early light environment does not affect overall retinal rod, cone, bipolar or ipRGC cell function in C57BL/6J mice.

In contrast and as expected, CD1 albino mouse retinas were significantly affected by perinatal light environment. LL-reared CD1 mice had significantly reduced a-wave (*F*_(2, 159)_ = 21.097, *p*<0.001; Figure 1B) and b-wave (*F*_(2, 159)_ = 42.392, *p*<0.001; Figure 1D) amplitudes of the dark-adapted ERG, compared to LD- and DD-reared mice, specially at higher light intensities. LL-reared mice had also a significantly reduced b-wave amplitude of the photopic ERG, compared to mice LD- and DD-reared mice, while LD-reared mice had a significantly smaller b-wave amplitude than DD-reared mice (*F*_(2, 538)_ = 770.096, *p*<0.001, Fig. 1F). Pupil response was also affected by postnatal light environment (*F*_(2, 10)_ = 7.651, *p* = 0.010) with LL-reared CD1 mice displaying a significantly bigger pupil area in response to the light stimulus compared to LD-reared mice (Fig. 1H). These results show that postnatal light environment, and specifically LL-rearing, causes a large reduction in the retinal function of albino CD1 mice, including their non-photic visual responses.

**Figure 2:**
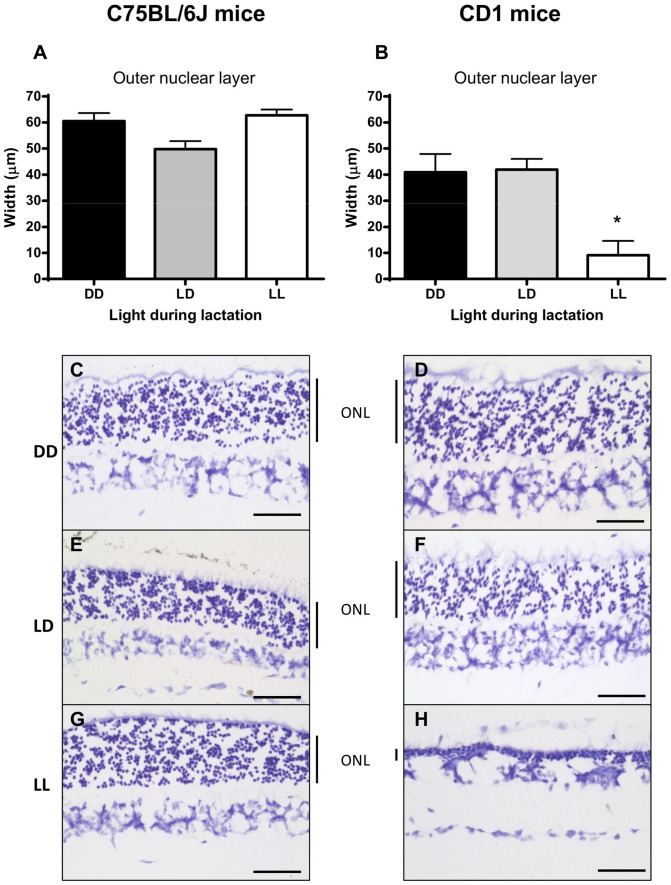
Postnatal light effects on retinal morphology in C57BL/6J and CD1 adult mice. Retinal morphology of adult mice reared in DD, LD or LL postnatally and later exposed to LD. (A, B) Outer nuclear layer width. (C-H) Photomicrographs of vertical sections through the retina. ONL – outer nuclear layer; DD – constant darkness; LD –24 h light-dark cycles; LL – constant light. *P<0.05 DD, LD vs. LL. Scale bar  = 50 µm. Data are presented as mean ± s.e.m. (n = 7).

We also assessed changes in retinal structure histologically by using cresyl violet staining and measuring the width of the ONL, which contains the nuclei of rods and cones, and is a good marker of retinal damage [30], [34]. We found no statistically significant differences in the width of the ONL due to early light environment in C57BL/6J mice (Figs. 2A, C, E, G), which suggests that early light environment does not affect the structural integrity of C57BL/6J mice retinas. In contrast, we found that postnatal light experience had a significant effect on ONL width (*F*_(2, 10)_ = 11.202, *p* = 0.003; Fig. 2B), with LL-reared CD1 mice showing a significant decrease in ONL width compared to LD- and DD-reared mice (Figs. 2B, D, F, H). Therefore, it appears that LL-rearing causes significant damage to the rod and cone nuclear layer in CD1 mice.

In summary, the combination of the ERG, PLR and histological data strongly indicates that exposure of CD1 mice to a LL environment during the first 3 weeks after birth will cause significant functional and morphological damage to their retinas, which will be carried into adulthood. In contrast, C57BL/6J retinas appear largely unaffected by postnatal light environment.

### Effects of chronic light exposure on the adult retina

**Figure 3:**
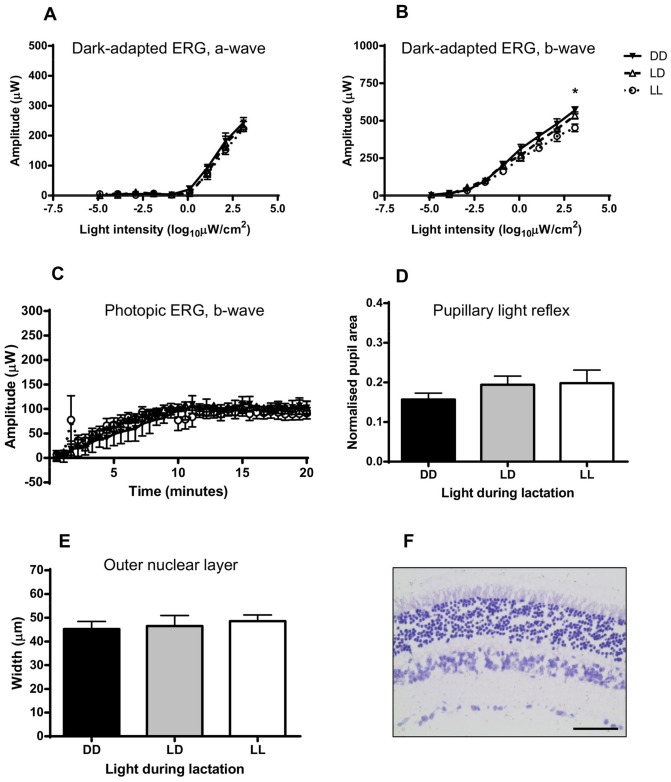
Postnatal light effects on retinal adaptation to chronic environmental light exposure in C57BL/6J adult mice. Retinal function and morphology of adult mice reared in DD, LD or LL postnatally and later exposed to LL. (A) Representative dark-adapted ERG a-wave amplitude. (B) Amplitude of the dark-adapted ERG b-wave. (C) Amplitude of the photopic ERG b-wave. (D) Pupillary light reflex. (E) Outer nuclear layer width. (F) Representative photomicrograph of a vertical section through the retina. ERG – electroretinogram; DD – constant darkness; LD –24 h light-dark cycles; LL – constant light; ONL – outer nuclear layer. *P<0.05 DD vs. LL. Scale bar  = 50 µm. Data are presented as mean ± s.e.m. (n = 5).

As we found in the previous experiment and also found by others [30], pigmented rodents are resistant to retinal damage by light. Nevertheless, pigmented animals are not totally immune to light damage when the light stimulus is strong enough [35]. Here, we tested whether chronic exposure to light (6 weeks LL) in adulthood induces retinal damage to C57BL/6J mouse retinas, and whether postnatal light experience affects the susceptibility of these mice to develop retinal damage. To test this, we assessed retinal function and structure in C57BL/6J mice exposed to LL (330 µW/cm^2^) for 6 weeks starting from P64, and finally re-entrained to 24 h LD cycles for a minimum of 3 weeks. We found that the dark-adapted ERG a-wave (*F*_(1,243)_ = 11.27, *p* = 0.001; Figs. 1A, 3A) and b-wave amplitudes (*F*_(1,241)_ = 8.075, *p* = 0.005; Figs. 1C, 3B), photopic ERG b-wave amplitude (*F*_(1,938)_ = 971.530, *p<*0.001; Figs. 1E, 3C), pupil response (*F*_(1,20)_ = 15.498, *p* = 0.001; Figs. 1G, 3D) and ONL width (*F*_(1,16)_ = 15.010, *p* = 0.001; Figs. 2A, 3E) were significantly decreased after LL exposure, suggesting a damaging effect of chronic light exposure on the adult C57BL/6J mouse retina. Interestingly, postnatal light environment did not appear to affect any of the variables measured: dark-adapted ERG a-wave amplitude (Fig. 3A), photopic ERG b-amplitude (Fig. 3C), pupillary light reflex (Fig. 3D), or outer nuclear layer width (Figs. 3E and 3F). We found a significant interaction in dark-adapted ERG b-amplitude, showing that LL-reared mice had a significantly lower b-wave amplitude than DD-reared mice, but this effect was only observed at the highest light intensity tested (*F*_(2,10)_ = 6.746, *p = *0.014; Fig. 3B).

Taken together, these data show that exposure of adult C57BL/6J mice to LL for a prolonged duration can slightly reduce retinal function and affect retinal structure, but without causing any major damage to the retina. Furthermore, in most cases all mice were affected in the same way, regardless of the light environment in which they were raised, indicating that postnatal light experience does not affect the later susceptibility of the animal to suffer retinal damage after light exposure.

### Effects of postnatal light environment on the chronic responses of the adult animal to light

**Figure 4:**
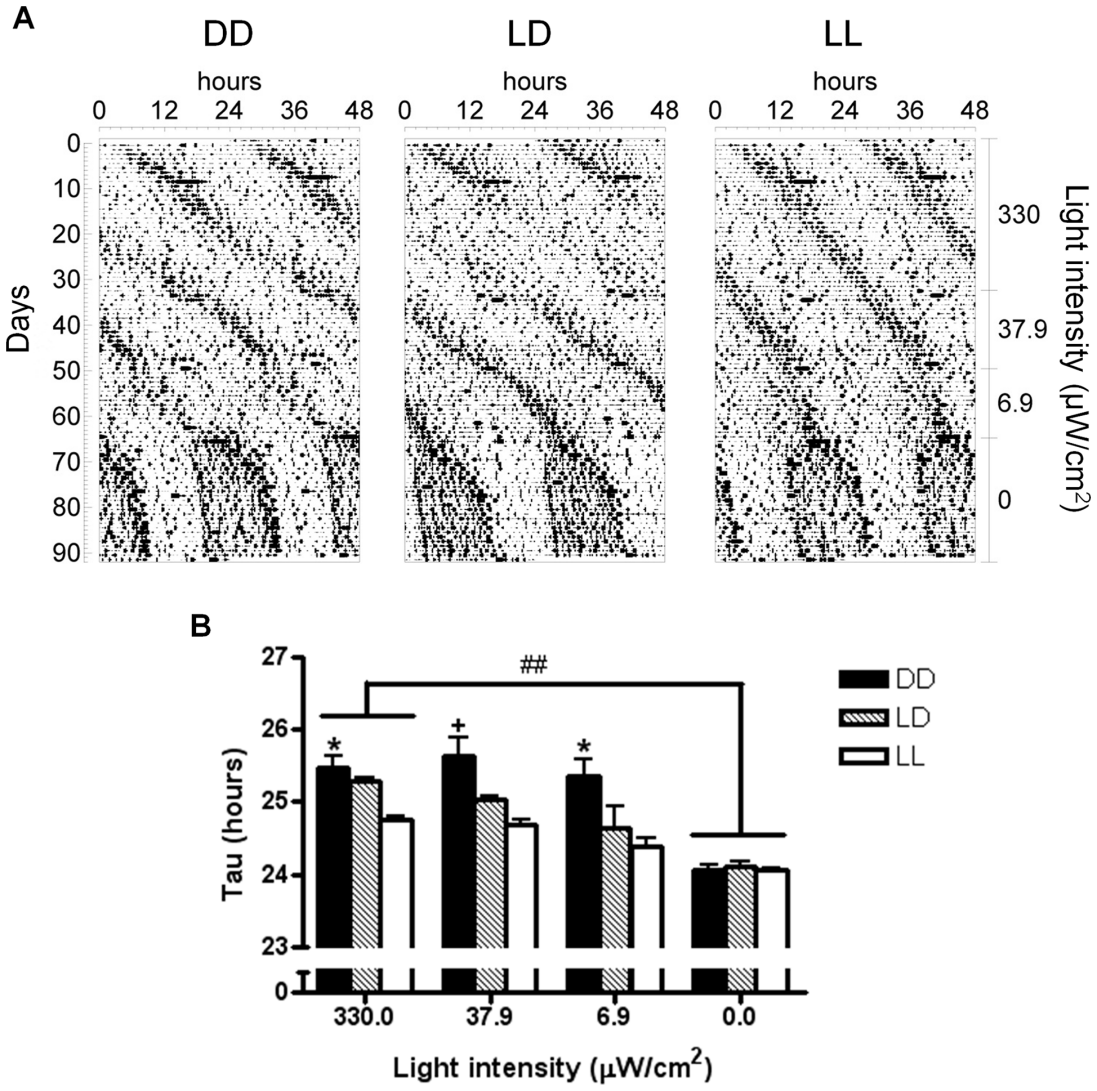
Postnatal light effects on circadian behavioral responses of adult C57BL/6J mice to chronic light exposure. (A) Representative double-plotted actograms showing the daily motor activity pattern of adult mice reared in DD, LD or LL postnatally (indicated on top of each actogram) and later exposed to LL of decreasing intensities (330.0 µW/cm^2^; 37.9 µW/cm^2^; 6.9 µW/cm^2^) and finally exposed to DD (0.0 µW/cm^2^). (B) Free-running period (tau) of the circadian rhythm of locomotor activity in each experimental stage. DD – constant darkness; LD –24 h light-dark cycles; LL – constant light. Main statistical effects: tau in DD vs. tau in LL 330.0 µW/cm^2^ (## p<0.001); whithin each light intensity, tau LL-reared mice vs. tau DD-reared mice (*P<0.05,+P = 0.05). Significant interaction: in LL-reared mice, tau 330.0 µW/cm^2^ vs. tau 6.9 µW/cm^2^ (p<0.001, not shown). Data are presented as mean ± s.e.m. (n = 5).

Previous studies on albino rats and mice have demonstrated that postnatal light experience impacts the future circadian behavior of the animals under a constant light environment, so that LL-reared animals will display a shorter free-running period (tau) in the locomotor activity rhythm compared to LD- and DD-reared animals when exposed to LL in adulthood [8], [9]. These responses could, in part, be explained by an impaired retinal function in LL-reared albino mice. Therefore, we set out to determine the specific role of postnatal light experience on the future circadian behavioral responses of the adult animal to LL in pigmented C57BL/6J mice, by exposing them to LL of various intensities (330 µW/cm^2^, 37.9 µW/cm^2^ and 6.9 µW/cm^2^). We found that at all intensities of LL, the tau of the circadian rhythm of locomotor activity was always shorter in LL-reared mice compared to DD-reared mice (*F*_(2, 33)_ = 32.465, *p*<0.001; Figs. 4A and 4B). We found a significant interaction between light intensity and light during lactation, so that only in LL-reared mice, the tau at the lowest intensity was shorter than that at the highest light intensity (*F*_(3, 14)_ = 20.716, *p*<0.001). We have previously shown that postnatal light environment does not affect gross retinal function and structure in C57BL/6J mice, therefore the difference between these two groups may be found instead at the SCN level, and specifically, in how the SCN processes photic input.

We then exposed all mice to DD (0.0 µW/cm^2^), to test their free-running rhythms in the absence of light. As expected, the average taus for all mice in DD were significantly shorter than those in LL of the highest intensity (F_(3,33)_ = 40.916, p<0.001, Figs. 4A and 4B). Interestingly, we found no differences due to perinatal light environment in any of the variables characterizing the circadian rhythm locomotor activity in DD, suggesting that, in the absence of light, overall clock function is similar between the groups. Taken together, these findings suggest that the adaptation of the circadian clock to light, but not its intrinsic function is dependent on postnatal light experience.

### Effects of postnatal light environment on the acute responses of the adult animal to light

**Figure 5:**
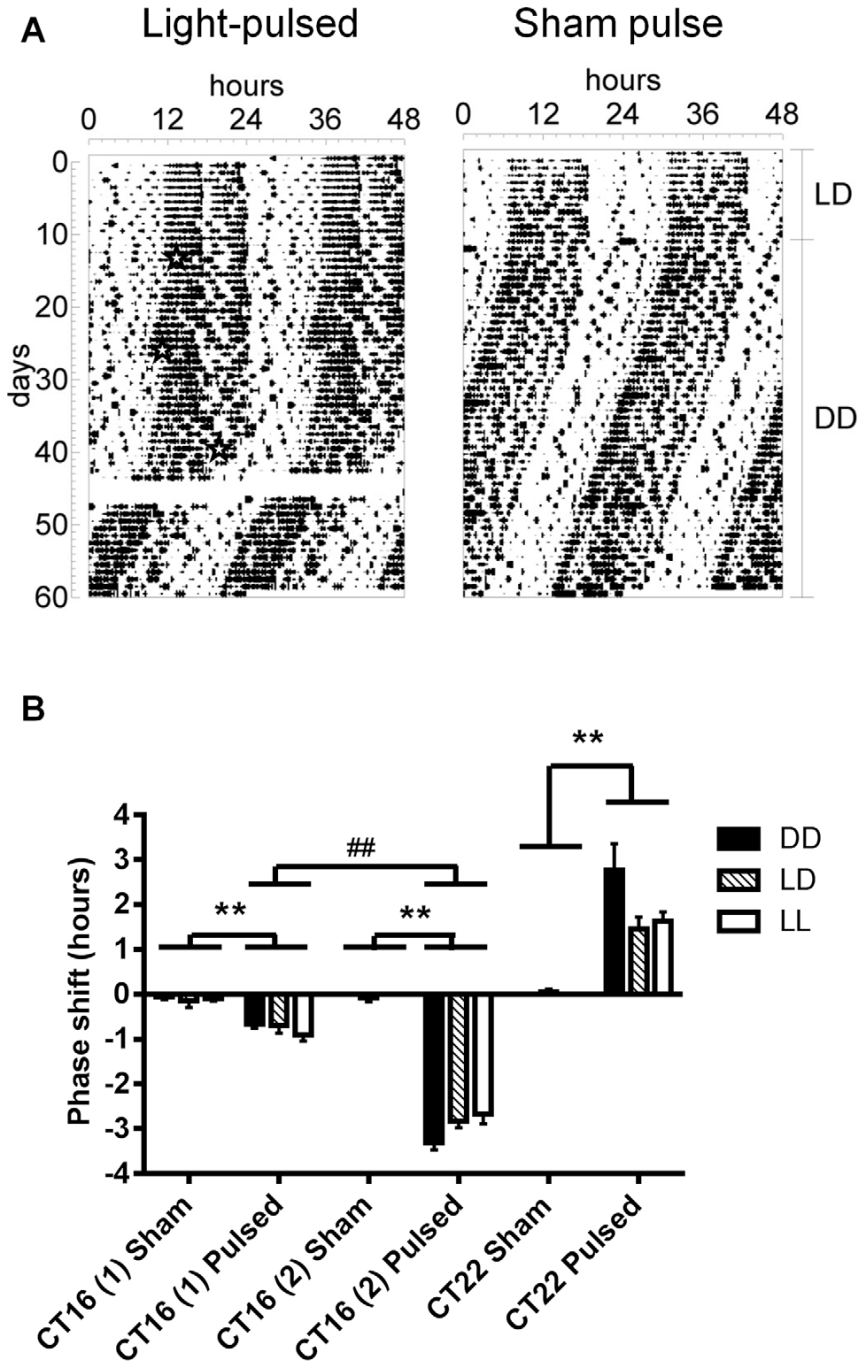
Postnatal light effects on circadian behavioral responses of adult C57BL/6J mice to acute light exposure. (A) Representative double-plotted actograms showing the daily motor activity pattern of adult mice that were given a light pulse (pulsed, indicated by a star) or sham pulse (non-pulsed) on a constant darkness background, at various times: CT16 (1), CT16 (2), and CT22. (B) Phase shifts in the circadian rhythm of locomotor activity of adult mice reared in DD, LD or LL postnatally and later exposed (Pulsed) or not (Non-pulsed) to a light pulse at various circadian times. CT – Circadian Time; DD – constant darkness; LD –24 h light-dark cycles; LL – constant light; SCN – Suprachiasmatic Nucleus. Main statistical effects: pulsed vs. non-pulsed (**P<0.01); dark-adapted –CT16(2)- vs. non-dark-adapted –CT16(1)- (##p<0.01). Data are presented as mean ± s.e.m. (n = 5–12).

As demonstrated earlier, the adaptive responses of the circadian clock to chronic light exposure are imprinted by postnatal light experience. Aschoff's rule states that tau increases with light intensity in nocturnal animals [36]. Therefore, it is possible that DD-reared mice are more sensitive than LL-reared mice to exposure to the same light stimulus, and thus display a longer tau in LL. To test whether DD-reared mice are indeed more sensitive to light than LL-reared mice, we examined the behavioral responses of these animals to phase-delaying and phase-advancing light pulses, as these have been shown to involve different mechanisms within the SCN [37], [38], [39]. We thus exposed adult C57BL/6J mice to various light pulses (30 min duration, 330 µW/cm^2^) on a dark environment, and measured their behavioral responses. High light intensity pulses have been shown to saturate behavioral phase shift responses [40], thus potentially masking differences between groups. Therefore, we here used the same light intensity for the pulses (330 µW/cm^2^) than that we had used in the retinal function and chronic LL experiments, thus a light intensity to which DD- and LL-reared mice responded similarly at the retinal level, but differently at the behavioral level. We found that all mice that had received a light pulse at CT16, independently on whether they were dark-adapted (second pulse) or not (first pulse) showed a phase delay which was significantly longer than the shift seen in mice that had received a sham pulse (*F*_(1, 45)_ = 335.275, *p*<0.01 for dark-adapted, *F*_(1, 45)_ = 32.915, *p*<0.01 for non-dark-adapted; Figs. 5A and 5B). As expected [41], dark-adaptation induced a larger phase delay (*F*_(1, 58)_ = 308.508, *p*<0.01; Fig. 5A and 5B). Nevertheless, there was no difference in the size of phase delay due to early light environment. When exposed to a light pulse at CT22, all mice responded with a phase advance, as opposed to sham-treated mice (*F*_(1, 44)_ = 29.048, *p*<0.01; Figs. 5A and 5B). But again, we found no difference in the size of the phase shift due to early light environment.

We then investigated the activation of the SCN after a light pulse at CT16, by examining phosphorylated ERK (pERK) and cFOS expression in the SCN after a 30-minute light pulse at CT16. pERK has been shown to play an important role in phase shifting after light pulses [42], [43], and it is one of the first elements in the SCN photic signaling pathway to be activated [44], [45]. *cFos* gene is upregulated in response to light pulses [46], and is commonly used as a marker of photic activation in the SCN. As expected [10], [46], we found that the light pulse induced a significant increase in the number of cFOS positive cells in the SCN, but no differences due to postnatal environment were found (mean ± SD  = 2,511.63±500.61 positive nuclei/mm^2^ for DD-reared mice; 2,370.80±350.59 positive nuclei/mm^2^ for LD-reared mice; 2,298.17±426.30 positive nuclei/mm^2^ for LL-reared mice). We obtained similar results with pERK immunostaining, with a significant increase in immunostaining density after the light pulse (*F*_(1, 41)_ = 59.458, p<0.001), but no differences due to postnatal environment (mean ± SD  = 20.40±7.16 a.u. for DD-reared mice; 19.89±4.37 a.u. for LD-reared mice; 21.60±8.21 a.u. for LL-reared mice). Taken together, these results suggest that perinatal light environment does not grossly imprint the acute photic signaling pathways within the SCN.

### Effects of postnatal light environment on future clock gene expression

Ciarleglio et al. [13] found that perinatal photoperiod imprints *Per1* clock gene expression in the SCN. We therefore used PER2::LUC mice here to examine clock gene imprinting by DD and LL postnatal light environments. We raised PER2::LUC mice under LD, LL or DD environments from P0 to P21. They were all then moved to LD until culled in adulthood (2–6 months of age). To avoid effects of time of day of culture preparation on the PER2::LUC rhythm [23], all mice were culled at ZT4-5.

**Figure 6:**
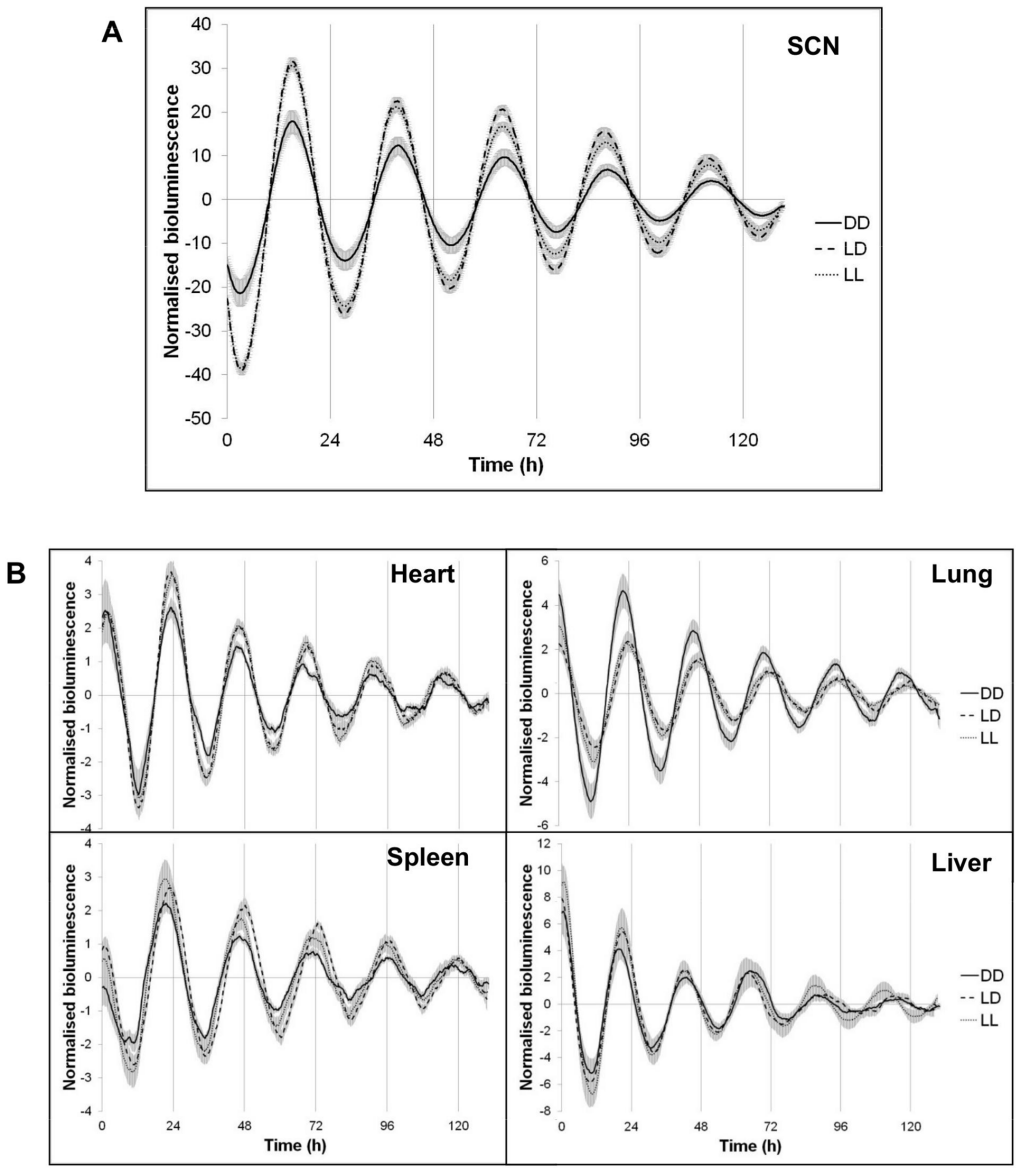
Postnatal light effects on the circadian rhythm of PER2::LUC expression in adult mice. (A–B) Representative time series of PER2::LUC rhythms displayed by SCN and peripheral oscillators of adult mice that were raised in DD, LD or LL postnatally and later exposed to LD. DD – constant darkness; LD –24 h light-dark cycles; LL – constant light. Data are presented as mean ± s.e.m. (n = 10–12).

Importantly, we found that the amplitude of PER2::LUC rhythms in the SCN was significantly lower in DD-reared mice compared to LD- and LL-reared mice (*F*_(2, 19)_ = 6.193, *p* = 0.008; Fig. 6A). We found no differences in any other variable measured in the SCN.

We also prepared organotypic cultures of peripheral tissues (heart, lung, liver, spleen), in order to test the imprinting of perinatal light environment on peripheral clocks. We found that PER2::LUC rhythm in the heart of DD-reared mice had a significantly lower amplitude compared to LL-reared mice (*F*_(2, 21)_ = 4.593, *p* = 0.022; Fig. 6B). In contrast, we found that DD-reared mice had a significantly higher amplitude of PER2::LUC rhythm in the lung compared to LL-reared mice (*F*_(2, 25)_ = 5.165, p = 0.013; Fig. 6B). In the spleen, we found differences in the phase, so that the peak of the PER2::LUC rhythm occurred significantly earlier in LL-reared mice compared to LD-reared mice (*F*_(2, 30)_ = 4.006, p = 0.029; Fig. 6B). In the liver, we found no differences in PER2::LUC rhythm due to early light environment (Fig. 6B), which could be due to the fact that our mice were fed *ad libitum*, and the liver's main environmental synchronizer is food [47]. Taken together these results demonstrate, for the first time, an imprinting effect of postnatal light environment on peripheral organs outside the SCN clock, and therefore suggest broad physiological consequences for the animal.

## Discussion

Here we applied a multi-level approach to investigate the extent of the programming effects of postnatal light experience on the circadian system. The data revealed significant imprinting effects on the circadian clock's adaptive responses to chronic light exposure, but not on gross retina function. In addition, we found evidence of programming in circadian peripheral clocks. These results substantially broaden our picture of how circadian system function is shaped by postnatal environmental experience.

### Effects of postnatal light environment on adult mouse retinal function and structure

We found that LL-rearing had a significant damaging effect on the retinas of albino mice, which was carried into adulthood. Due to lack of pigment, albino mouse retinas are particularly sensitive to damage by light, including exposure to LL, compared to pigmented mice [29], [30], [34]. In addition, LL-rearing has been shown to prevent the developmental increase in the number of ipRGCs in the retinas of albino CD1 mice but not pigmented C3H/He mice [48], [49], suggesting that LL-rearing may impact circadian function of these mice later in life.

In line with the results of González-Menéndez et al. [48], we found no gross alteration of retinal function or morphology in LL-reared pigmented C57BL/6J mice, suggesting that pigmented mouse retinas are resistant to LL exposure during postnatal development. We did, however, observe a slight decrease in retinal function and ONL width when C57BL/6J mice were exposed to LL for 6 weeks in adulthood. Nevertheless, it did not cause a major impairment to their retinal responses to light, and interestingly, it was independent on postnatal light environment. These results suggest that postnatal light experience does not affect the later susceptibility of the retinas to damage by long-term light exposure.

### Effects of postnatal light environment on the behavioral responses of the adult animal to light

Previous experiments in albino mice have shown that, in contrast to LD- and DD-reared animals, those raised in LL will display a shorter free-running period (tau) when exposed to LL in adulthood [8]. We here exposed pigmented C57BL/6J adult mice to LL and found similar results: LL-reared mice showed shorter taus of the locomotor activity rhythm compared to DD-reared mice. Melanopsin-knockout mice display shorter taus in LL compared to wild type mice [50], so in principle, an impairment of melanopsin-ipRGC function in LL-reared mice could explain our results. However, we found that PLR responses, which have been shown to reflect melanopsin-driven (ipRGC) function [33], are similar between all the groups. This suggests that melanopsin function is largely spared in our mice, and therefore, the observed differences in tau in LL between DD- and LL-reared mice do not originate at the retinal level.

We then tested the sensitivity of the circadian system of our mice to light by examining their behavioral responses to acute light pulses, and found that these were independent of postnatal light experience. These results are supported by the finding that clock photic responses are also similar between the groups, as indicated by similar levels of induction of pERK and cFOS expression in the SCN after a phase-delaying light pulse (this study, [10]). We did, however, not obtain a full phase response curve, and therefore it is possible that we may have missed changes in the shape of this curve that could explain the different taus in LL [51]. Nevertheless, different mechanisms are thought to explain the phasic and tonic responses of the SCN clock to light [52], and thus it is possible that postnatal light environment differently imprints the long-term tonic and the short-term phasic responses of the circadian clock to light.

We found no differences in locomotor activity rhythm in the absence of light (DD) due to early light environment. This is different to what has been found in Wistar albino rats, where DD-reared animals show stronger rhythms than LL-reared rats even after enucleation [53]. This could be, in part, because of an impaired retinal input to the SCN prior to enucleation in albino rats. Indeed, an intact retinal input during development has been found to be critical for normal SCN morphology and function [54], [55]. Our results are also different to those of Ciarleglio et al. [13], who found that perinatal photoperiod affected the tau and duration of activity of the locomotor activity rhythm for the first 3 days in DD. This could be explained by the fact that the environmental manipulation performed by Ciarleglio et al. (photoperiod) is different to that in our study (LL- and DD-rearing). Here we exposed mice to DD for 4 weeks after LL, to ensure minimal aftereffects of the previous light exposure [56], [57], and found no differences in the waveform of the locomotor activity rhythm due to postnatal light environment. However, we have observed differences in the locomotor activity waveform due to postnatal light experience when mice are exposed to 24 h LD cycles [7] and LL (this study). Taken together, these results further strengthen the idea that our environmental light manipulations during postnatal development may imprint chronic adaptation of the circadian clock to light, but not intrinsic clock function in the absence of light.

### Effects of postnatal light environment on future clock gene expression

Exposure of adults to LL results in the loss of the circadian rhythms of locomotor activity [58], [59], [60], plasma melatonin [60], sex hormones [61] and body temperature [58], [59], which correspond with weakened or lost oscillations in the SCN [56], both at the neuronal (firing rate [62]) and molecular clock levels (expression of mPER2 clock protein [63]). The cause of this decline in circadian amplitude at the tissue and electrical activity levels appears to be a decrease in SCN cell population synchronization rather than an impairment of the individual SCN cell rhythm-generating ability [64]. In line with this, Ohta et al. [65] showed that LL-reared *Per1*:GFP mice had robust SCN rhythms at the cellular level but not at the whole tissue level. In addition, *Per1*:GFP mice showed lower amplitude locomotor activity rhythms when exposed to LL after weaning. However, we and others have consistently found increased amplitude behavioral rhythms in LL-reared rats [11], [15] and mice (this study, [8]) when exposed to LL in adulthood, which suggests that LL exposure during development may confer the circadian system with a certain resilience to the disrupting effects of LL later in life. Although further work is required to identify the mechanisms behind these effects, one possible explanation is a long-term increase in intra-SCN synchronization in LL-reared animals. This is supported by our finding that LL-reared mice show higher amplitude PER2::LUC rhythms at the SCN level, as increased amplitude of the SCN output has been linked to an increase in synchronization of the SCN neural population [56]. Astrocytes may play an important part in the synchronization of SCN oscillators as they have been shown to facilitate photic signal transmission within the SCN [55], [66], and may also be involved in SCN time-keeping function [67], [68], [69]. Furthermore, they appear to be key for the regulation of locomotor activity behavior in LL [70], and their number and shape is altered in LL-reared mice [12]. Taken together these data strengthen the hypothesis that postnatal light environment may affect the level of SCN synchronization, and as a consequence, the adaptive responses of the SCN to future light environments.

A novel and interesting finding of the present study is that the effects of postnatal light environment are not restricted to the SCN. Previous studies demonstrated changes in neuropeptide and early-gene expression in several hypothalamic areas in LL- and DD-reared mice [7], [10], which suggested further-reaching effects of postnatal light environment. We here examined PER2::LUC rhythm in the heart, lung and spleen, and found long-term effects of early light experience either at the amplitude or phase levels. Cellular oscillations in peripheral tissues are synchronized at the organism level by the SCN [71], which suggests that imprinting of SCN function by postnatal light experience may subsequently impact on peripheral clock function. Although the entraining mechanisms for peripheral oscillators are still unknown, regulation of activity, feeding and body temperature rhythms by the SCN seem to play an important role [72], [73]. It is difficult to predict what are the functional consequences of altered clock gene expression within an organ, but clock gene rhythms in the heart have been linked to changes in heart rate and blood pressure [74], in the lung it is thought they affect the severity of airway inflammation in asthma patients [75], while in the spleen they are involved in the immune response [76]. Therefore, alterations in the expression of clock genes could affect the normal physiology of these tissues, and thus potentially have health consequences.

## Conclusions

LL exposure in adulthood leads to arrhythmic behavioral, physiological and neuronal patterns. However, and in stark contrast, LL-rearing appears to confer a certain resilience to the SCN, so that when an animal is exposed to LL or even just normal 24 h LD cycles later in life, it will show higher amplitude behavioral and clock gene circadian rhythms, possibly as a consequence of an increased synchronization within SCN cell populations. The opposite seems to apply to DD-reared mice. This is important, as reduced synchrony and SCN amplitude output are believed to have profound consequences on the individual's health [73], [77], [78]. In addition, our results indicate that this SCN reorganization may affect the regulation of circadian rhythms in peripheral oscillators, which suggests broad consequences for overall circadian regulation in an organism. Uncovering the specific mechanisms imprinted by early light experience is the first critical step towards elucidating the long-term consequences of circadian rhythm programming on the individual's future health and wellbeing.
